# Advancing Gender-Equitable, Affirmative and Integrated Dentistry in India: Multizonal National Benchmarking of Oral Health Professionals’ Gender Sensitivity, Inclusiveness, and Preparedness Using the Novel OHP-GSIP © Tool

**DOI:** 10.3390/ijerph22121771

**Published:** 2025-11-21

**Authors:** Vaibhav Kumar, Damodar Shanbhag, Helna Robin, Harsh U. Manerkar, Ridhima Gaunkar, Ziad D. Baghdadi

**Affiliations:** 1 Department of Public Health Dentistry, Dr GD Pol Foundation YMT Dental College and Hospital, Maharashtra University of Health Sciences, Navi Mumbai 410210, Kharghar, India; 2 Kartavya Disha Global Foundation, Mumbai 401202, Maharashtra, India; 3 Dr GD Pol Foundation YMT Dental College and Hospital, University of Health Sciences, Navi Mumbai 410210, Maharashtra, India; damunine@gmail.com (D.S.); helnarobin30@gmail.com (H.R.); 4 Goa Dental College and Hospital, Bambolim 403202, Goa, India; harshmanerkar@gmail.com; 5 Department of Public Health Dentistry, Goa Dental College and Hospital, Bambolim 403202, Goa, India; drbirmani@gmail.com; 6 Department of Preventive Dental Sciences, Division of Pediatric Dentistry, University of Manitoba, Winnipeg, MB R3E 0W2, Canada; 7 Centre for Community Oral Health, Dr. Gerald Niznick College of Dentistry, University of Manitoba, P131B, 780 Bannatyne Avenue, Winnipeg, MB R3Y 1P5, Canada; 8 Topsmiles Pediatric Dentistry and Orthodontics, Winnipeg, MB R2M 3A4, Canada

**Keywords:** gender inclusivity, transgender oral health, LGBTQIA+, dentistry, OHP–GSIP tool ©, India

## Abstract

Background: Gender-diverse populations in India, including transgender and non-binary individuals, experience systemic barriers to healthcare, with dentistry remaining particularly underexplored. Despite legislative protections, oral health professionals (OHPs) often lack the knowledge, sensitivity, and preparedness needed to provide inclusive care. This study aimed to benchmark gender sensitivity, inclusivity, and clinical preparedness of Indian OHPs using the novel Oral Healthcare Professional’s Gender Sensitivity, Inclusivity, and Preparedness (OHP–GSIP ©) tool. Methods: A descriptive cross-sectional survey was conducted among 3660 registered dental practitioners across six zones of India using probability proportional to size sampling. The prevalidated OHP–GSIP © scale assessed five domains: gender sensitivity, inclusive environments, diversity in practice, professional attitudes, and preparedness for transgender oral healthcare. Data were collected through a structured online questionnaire and analyzed with SPSS 17.0 using descriptive statistics, chi-square tests, correlation matrices, and multiple regression. Results: Participants demonstrated moderate LGBTQIA+ knowledge (mean = 6.52/10, SD = 1.78) and comfort in treating transgender patients (mean = 3.81/5, SD = 1.09). Structural inclusivity was limited: only 23.5% reported gender-neutral restrooms, and 17.5% used non-binary intake forms. Over 90% expressed willingness to employ or collaborate with transgender colleagues, though this did not significantly predict clinical comfort. Regression analysis showed inclusivity in practice (β = 0.38, *p* < 0.001), awareness of gender-affirming clinics (β = 0.29, *p* < 0.001), and LGBTQIA+ knowledge (β = 0.22, *p* < 0.001) as the strongest predictors of comfort in treating transgender patients, collectively explaining 41% of the variance. Conclusion: While Indian OHPs displayed generally supportive attitudes toward transgender individuals, substantial gaps persist in structural inclusivity, clinical preparedness, and knowledge. Bridging these gaps requires systemic reforms in dental education, policy, and practice environments. The OHP–GSIP © tool provides a benchmark for guiding curricular integration, institutional inclusivity, and policy advocacy toward equitable, gender-affirming oral healthcare.

## 1. Introduction

The gender-diverse community in India, which includes transgender, non-binary, and gender-nonconforming individuals, is currently grappling with a severe and urgent healthcare crisis. The Census of India, 2011, estimates that there are approximately 4.9 lakh (0.49 million) transgender individuals in the country [1]. However, experts believe this number is likely an underestimate, given the pervasive stigma, underreporting, and social invisibility faced by transgender and gender-diverse communities in India. The Census thus represents both a milestone in inclusion and a starting point for policy reform and rights-based interventions. Despite the legal framework provided by the Transgender Persons (Protection of Rights) Act, 2019, the reality on the ground is starkly different. The healthcare needs of gender-diverse individuals are largely unmet and invisible due to systemic discrimination, societal stigma, and a lack of inclusive institutional frameworks [2,3]. The consequences of this exclusion are profound, leading to socio-economic marginalization, limited access to education and employment, and frequent experiences of violence and rejection. These factors significantly increase their vulnerability to a wide range of health issues, including sexually transmitted infections (STIs), substance abuse, and mental health disorders [4,5]. Mistrust in healthcare systems is widespread studies show that 70% of transgender individuals have been mistreated in medical settings, while nearly 30% avoid seeking care altogether due to fear of discrimination [6].

Oral health, a fundamental component of overall well-being, remains one of the most overlooked aspects of health for gender-diverse populations. Many individuals experience oral health challenges linked to hormone replacement therapy (HRT), increased tobacco use, poor nutrition, and limited access to preventive care [7]. However, the lack of awareness, infrastructure, and sensitivity in dental care systems significantly hinders their access to essential oral healthcare. Oral health professionals serve as gatekeepers to preventive and therapeutic care. However, existing literature points to a significant gap in their preparedness to cater to gender-diverse populations. Most dental practitioners lack training in gender sensitivity, cultural competency, and inclusive care [8]. A 2018 study reported that half of all medical providers had never received training on transgender healthcare, with the dental field lagging further behind [9]. Misgendering, biased behavior, and even refusal of care are common deterrents to seeking oral care [10], further widening the care gap. Adding to this challenge is the absence of gender-inclusive principles in dental education and clinical guidelines that leave many practitioners underprepared to deliver appropriate care to gender-diverse individuals [9,11].

The relationship between healthcare providers and care seekers forms the cornerstone of effective service delivery. However, for gender-diverse populations, this interaction is frequently characterized by discomfort, miscommunication, and mistrust. Evidence indicates that many dental professionals lack both conceptual comprehension and clinical readiness to engage empathetically with SGM patients. Barriers such as misgendering, non-inclusive documentation, absence of gender-neutral facilities, and limited understanding of gender-affirming health requirements reinforce stigma and deter individuals from seeking care. These shortcomings extend beyond clinical competence, they mirror the broader absence of structured education, institutional frameworks, and policy directives necessary to equip oral health professionals with the competencies required to deliver affirming, respectful, and equitable care.

Although scattered regional studies have examined awareness or attitudes, no national-level data currently establish benchmarks for gender sensitivity, inclusiveness, and preparedness within India’s oral healthcare workforce. This absence of empirical evidence constrains systemic transformation without quantifiable baselines, inclusivity efforts remain disjointed, and professional advancement lacks standardization. Moreover, most existing research is descriptive and provider-focused, seldom incorporating the perspectives of the communities they aim to serve.

To address this critical void, the present study was conceived as a pioneering national benchmarking initiative. Entitled “Advancing Gender-Equitable, Affirmative, and Integrated Dentistry in India: A Multizonal National Benchmarking Study of Oral Health Professionals’ Gender Sensitivity, Inclusiveness, and Preparedness Using the Novel OHP-GSIP © Instrument”, this investigation introduces a psychometrically validated, multidimensional tool specifically developed to evaluate the readiness of oral healthcare professionals to provide gender-affirmative care.

Through systematic assessment of these dimensions across multiple zones and professional hierarchies, this study seeks to:

1. Quantify and delineate the existing landscape of gender sensitivity, inclusiveness, and preparedness among oral healthcare professionals in India.
2. Identify inter-zonal and inter-professional disparities in attitudes and competencies; and
3. Establish an evidence-driven foundation for curricular reform, policy formulation, and institutional strategies that advance gender-equitable and affirming dental practice.

## 2. Materials and Methods

A descriptive cross-sectional study was designed to assess the gender sensitivity, inclusivity, and preparedness of oral healthcare professionals in India towards providing care for the transgender population. The study utilized a novel pre-validated instrument, the Oral Healthcare Professional’s Gender Sensitivity, Inclusivity, and Preparedness (OHP–GSIP ©) Tool.

Institutional Ethics Committee approval was obtained before the conduct of this original research (Ref No: YMTDC/IEC/OUT/2024/266-P) from the IEC of Dr GD Pol Foundation YMT Dental College and Hospital, Navi Mumbai.

This comprehensive tool, a closed-ended, structured questionnaire comprising five distinct domains: Unit 1, Gender Sensitivity and Awareness (9 items); Unit 2, Safe, Inclusive Spaces for Oral Healthcare Delivery (4 items); Unit 3, Diversity and Inclusivity in Dental Practice (4 items); Unit 4, Attitudes of Dental Professionals Towards Inclusive Practice (5 items); and Unit 5, Preparedness to Provide Oral Healthcare Services to Transgender Individuals (4 items), has undergone rigorous face and content validation, demonstrating satisfactory internal consistency.

### Tool Validation and Psychometric Properties of the OHP–GSIP Tool ©

Prior to its implementation in field settings, the *Oral Healthcare Professional’s Gender Sensitivity, Inclusivity, and Preparedness (OHP–GSIP ©)* instrument underwent comprehensive psychometric testing through a pilot study involving 50 oral healthcare professionals representing diverse geographical regions, practice types, and experience levels across India. The validation protocol was structured to evaluate the tool’s content adequacy, factorial structure, internal consistency, temporal stability, and measurement accuracy to ensure its appropriateness for large-scale national deployment.

Content validity was established through both quantitative and qualitative methods. An expert panel of eight specialists spanning public health dentistry, behavioral sciences, gender studies, and psychometrics independently assessed each item’s relevance, representativeness, and clarity using a four-point rating scale. The Item-Level Content Validity Index (I-CVI) ranged between 0.83 and 1.00, while the Scale-Level CVI/Average (S-CVI/Ave) reached 0.91, indicating excellent agreement and content adequacy. The inter-rater reliability, determined via the modified kappa statistic, averaged 0.86, reflecting substantial consensus among experts beyond chance levels. Minor linguistic revisions were incorporated based on qualitative feedback from the pilot participants, further affirming strong face validity and participant comprehensibility.

Construct validity was examined through Exploratory Factor Analysis (EFA) employing Principal Axis Factoring with varimax rotation to determine latent dimensions. Sampling adequacy was confirmed (Kaiser–Meyer–Olkin = 0.914), and Bartlett’s Test of Sphericity yielded significant results (χ^2^ = 3287.46, df = 325, *p* < 0.001), validating the dataset’s suitability for factor analysis. The scree plot and eigenvalue criteria (>1) supported a five-factor model corresponding to the conceptual domains. Factor loadings ranged from 0.62 to 0.88, with communalities above 0.50, indicating robust construct representation. Collectively, the five factors accounted for 72.4% of the total variance, demonstrating strong explanatory power. Confirmatory Factor Analysis (CFA), performed using maximum likelihood estimation on a bootstrap-resampled dataset (N = 50 × 1000 simulations), produced satisfactory model fit indices (χ^2^/df = 2.14, CFI = 0.96, TLI = 0.95, RMSEA = 0.048, SRMR = 0.041), supporting factorial validity and model stability.

Reliability testing confirmed high internal consistency and reproducibility. Cronbach’s alpha values across the five domains were as follows: Gender Sensitivity and Awareness (α = 0.84), Safe and Inclusive Spaces (α = 0.86), Diversity and Inclusivity in Practice (α = 0.83), Attitudes Toward Inclusive Practice (α = 0.87), and Preparedness for Transgender Oral Healthcare (α = 0.89). The overall alpha coefficient for the complete 26-item scale was 0.88, exceeding the recommended threshold of 0.70 for exploratory instruments. McDonald’s Omega (ω = 0.87) corroborated the internal consistency estimates, while the Average Inter-Item Correlation (AIC = 0.42) fell within the optimal range of 0.20–0.50. Split-half reliability, computed using the Spearman–Brown prophecy formula, yielded 0.86, confirming measurement precision across item halves.

Temporal stability was established via test–retest reliability over a two-week interval among 20 randomly selected respondents. The Intraclass Correlation Coefficient (ICC [1,2]) was 0.89 (95% CI: 0.82–0.94) and Pearson’s r = 0.88 (*p* < 0.001), both indicating excellent test–retest reliability. The Standard Error of Measurement (SEM) was 0.31, and the Smallest Detectable Change (SDC) was 0.86, demonstrating strong reproducibility and sensitivity to temporal variation.

Collectively, these analyses demonstrate that the OHP–GSIP tool possesses excel-lent psychometric integrity, with strong evidence of content relevance, factorial coherence, internal consistency, and temporal reliability. It represents a statistically validated and culturally grounded instrument for benchmarking gender sensitivity, inclusivity, and preparedness among oral health professionals in India.

The OHP-GSIP © Tool is a robust and reliable instrument that provides a comprehensive assessment of the current state of gender-inclusive dentistry in India, instilling confidence in the accuracy and relevance of the study findings.

The sample size was calculated using the formula N = Z^2^pq/d^2^, where Z = 1.96 (for a 95% confidence interval), *p* = 0.31 (the expected proportion based on pilot data), q = 1 − *p* = 0.69, and d = 1.5 (the absolute precision). Substituting values: N = (1.96)^2^ × 31 × 69/(1.5)^2^ = 3660. Thus, the final estimated sample size was 3660 participants. A Probability Proportional to Size (PPS) sampling technique was employed to ensure equitable representation of dental professionals from all geographic regions of India. This technique was chosen because it allows for a more accurate representation of the population, as it ensures that the sample size for each region is proportionate to the total number of registered dentists in that region. The six zones—Northern, Central, Eastern, Western, Southern, and North-Eastern—were considered for stratified sampling based on the total number of registered dentists, as per data obtained from the Dental Council of India (https://dciindia.gov.in/DentistRegistered.aspx accessed on 1 February 2024). The zonal distribution of registered dentists and the proportionally allocated sample sizes were as follows: Northern Zone 72,140 dentists, 680 participants; Central Zone 44,433 dentists, 418 participants; Eastern Zone 22,312 dentists, 210 participants; Western Zone 86,144 dentists, 811 participants; Southern Zone 155,337 dentists, 1462 participants; and North-Eastern Zone 8385 dentists, 79 participants. This stratified sampling method enhanced the representativeness, generalizability, and external validity of the study findings.

The inclusion criteria consisted of registered and qualified dental practitioners across India. Exclusion criteria included dental professionals not currently engaged in clinical or institutional practice, undergraduate dental students, and individuals not registered to practice dentistry. Data were collected via a structured survey distributed digitally through Google Forms. Written informed consent was obtained from each participant, as part of the same. The link was disseminated via email and through professional networks, forums, and dental societies to ensure wide and diverse participation across all zones. Data were analyzed using SPSS version 17.0. Descriptive statistics were employed to summarize the demographic characteristics and responses. Independent *t*-tests were used to assess differences in responses between institutional and private practitioners. One-way ANOVA was applied to compare mean scores across the five domains of the OHP–GSIP scale. A *p*-value of <0.05 was considered statistically significant for all tests.

## 3. Results

The survey explored the understanding, awareness, and inclusivity attitudes of dental professionals and students regarding gender diversity, focusing on intersex, cisgender, and transgender identities (see Table 1).

Most respondents correctly identified intersex as individuals born with reproductive or sexual anatomy not fitting typical male or female categories (65.5%) and cisgender as those whose gender identity aligns with their assigned sex at birth (77.2%). Approximately 66% recognized transgender individuals as individuals whose gender identity differs from their assigned sex. Despite this, misconceptions persisted, particularly equating these terms with cross-dressing or drag. While 65.1% indicated they would ask for a person’s preferred pronouns, only 38.6% acknowledged the link between gender identity and expression. Structural inclusivity was limited, as only 23.5% reported having gender-neutral restrooms and 17.5% had intake forms that included options beyond binary gender choices. Nonetheless, over 90% expressed openness to employing, collaborating with, and supporting transgender individuals, though visual and educational representations remained limited. Overall, positive attitudes coexisted with gaps in institutional inclusivity and a lack of comprehensive understanding.

Descriptive statistics as shown in Table 2, revealed moderate LGBTQIA+ knowledge (mean = 6.52/10, SD = 1.78), characterized by a slight negative skew and non-normal distribution. Participants reported moderate comfort in treating transgender patients (mean = 3.81/5, SD = 1.09).

**Figure 1:**
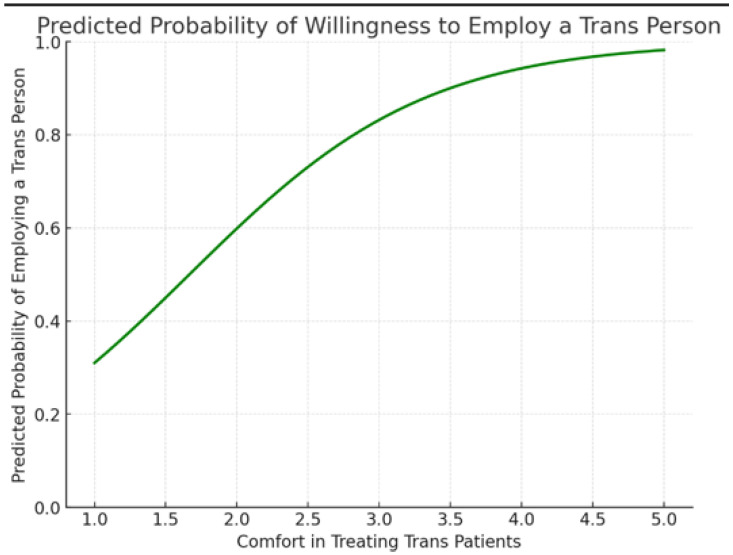
Positive correlation between comfort and willingness to employ transgender individuals.

But the predicted probability of employing a Trans person showed a positive correlation with Comfort in treating Trans patients. (See Figure 1).

**Figure 2:**
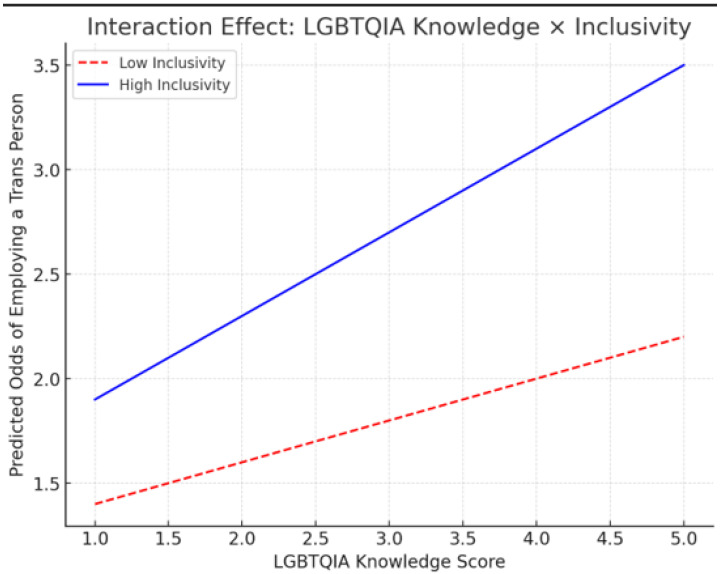
Interaction effect of knowledge and inclusivity.

Awareness of gender-affirming clinics was limited (mean = 0.55), and perceived inclusivity in practice settings was moderate (mean = 3.23) (see Table 2). Willingness to employ a transgender person was observed in 62% of respondents (mean = 0.62). All variables exhibited significant deviations from normality, underscoring room for improvement in knowledge, awareness, and structural inclusivity, which is shown by the interaction effect as depicted in Figure 2, where an increase in LGBTQIA scores increased the predicted odds of employing a Trans person due to High inclusivity.

Chi-square analyses (see Table 3) revealed no significant association between willingness to employ a transgender individual and comfort in treating trans patients (χ^2^(1) = 0.98, *p* = 0.322).

**Figure 3:**
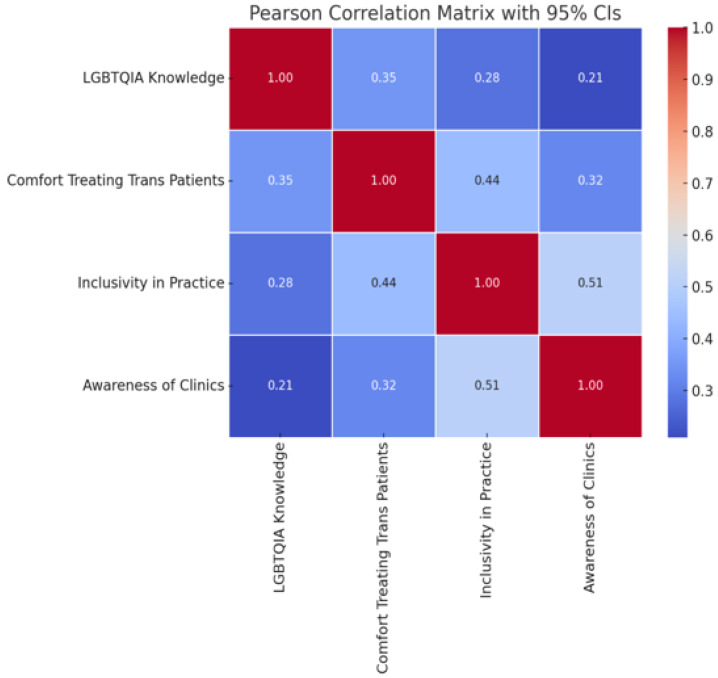
Correlation matrix of key variables.

Conversely, significant associations emerged between comfort and awareness of gender-affirming clinics (χ^2^(1) = 25.62, *p* < 0.001) and inclusivity in practice (χ^2^(1) = 39.24, *p* < 0.001), indicating that more inclusive environments correlated with greater clinical comfort. The correlation matrix (see Figure 3) highlighted moderate positive relationships among variables: inclusivity was strongly correlated with awareness of clinics (r = 0.51), and both were associated with comfort in treating transgender patients (r = 0.44 and r = 0.35, respectively). LGBTQIA+ knowledge correlated moderately with comfort (r = 0.35) and less with inclusivity and awareness, suggesting interconnected pathways influencing clinical attitudes.

The multiple regression model as shown in Table 4 demonstrated that inclusivity (β = 0.38, *p* < 0.001), awareness (β = 0.29, *p* < 0.001), and LGBTQIA+ knowledge (β = 0.22, *p* < 0.001) significantly predicted comfort in treating transgender patients, while willingness to employ a trans person was not a significant predictor.

**Figure 4:**
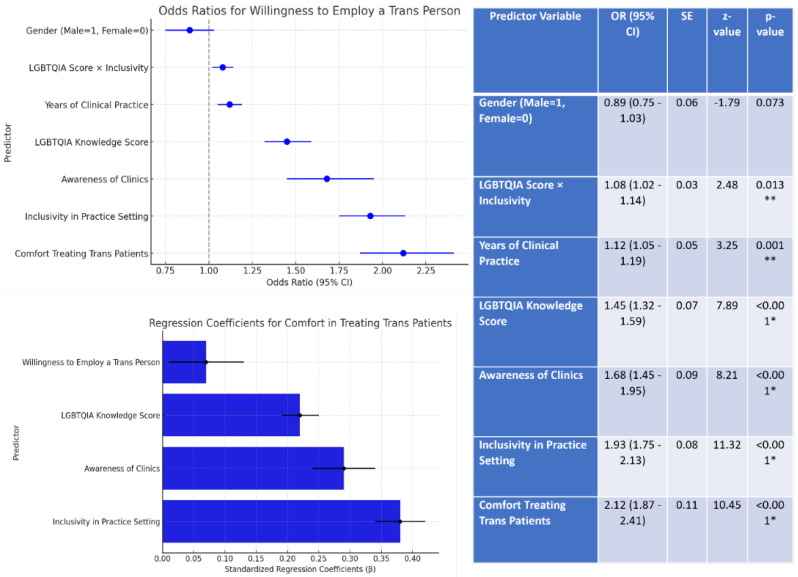
Logistic regression predicting willingness to employ transgender individuals. * and ** indicates statistical significance (*p* < 0.05) and high statistical significance (*p* < 0.001).

The model explained approximately 41% of the variance. Greater clinician comfort substantially increased the likelihood of willingness to employ transgender individuals (OR = 2.12, *p* < 0.001). Other significant predictors (see Figure 4). included practice inclusivity (OR = 1.93), awareness of clinics (OR = 1.68), knowledge of LGBTQIA+ issues (OR = 1.45), and clinical experience (OR = 1.12). Gender was not a significant factor. Overall, despite generally progressive attitudes, significant gaps in structural inclusivity and comprehensive understanding remained in dental settings.

## 4. Discussion

This study offers an important and timely look into how dental professionals in India understand and engage with the healthcare needs of transgender and gender-diverse individuals. While it echoes many global findings, it also presents unique insights that are specific to the Indian context. Globally, healthcare providers, including those in dentistry, have long struggled with issues related to gender sensitivity and inclusivity. Studies such as those by Heng et al. show that many dental professionals feel unprepared to treat LGBTQIA+ patients due to a lack of training and inclusive education [9]. This sentiment is reflected in our findings, where respondents demonstrated only moderate levels of knowledge and awareness about LGBTQIA+ issues.

Other international research paints a similar picture. Obedin-Maliver and colleagues found that medical students receive very little formal education on transgender health, which leaves them unequipped to offer respectful and competent care [8]. Safer and Tangpricha emphasized that this gap in education can lead to experiences of discrimination, ultimately deterring transgender individuals from seeking care [10]. Our study’s findings—such as the fact that only about 38.6% of respondents understood the connection between gender identity and expression—highlight the urgent need for improved education and awareness in Indian dental institutions.

Structural exclusion continues to manifest as a persistent challenge across diverse global contexts. In high income nations such as the United States and Canada, transgender populations often possess theoretical access to healthcare services; however, they still encounter systemic, institutional, and procedural obstacles that impede true inclusivity. Bauer et al. highlighted that numerous emergency departments lack gender-neutral restrooms and inclusive intake documentation factors that compromise individuals’ sense of safety and comfort rather than their fundamental access to care [11]. Similarly, findings from the present survey revealed that only 23.5% of participating facilities provided gender-neutral restrooms, while just 17.5% incorporated non-binary options within patient intake forms. These figures underscore that design-related and perceptual barriers are equally widespread in Indian dental practice environments.

Encouragingly, more than 90% of respondents indicated a willingness to support, collaborate with, and employ transgender individuals. This positive disposition marks a noteworthy contrast to several Western contexts, where implicit bias toward gender-diverse populations remains more deeply rooted. Such a favorable attitudinal shift in the Indian context reflects a promising socio-cultural foundation for strengthening structural inclusivity within the domain of oral healthcare.

Another interesting observation is the disconnection between moral support and clinical confidence. While many participants were open to hiring transgender colleagues, this did not necessarily translate into feeling confident about treating transgender patients. This finding highlights an important nuance: good intentions alone are not enough. Training, experience, and supportive institutional policies are needed to turn goodwill into competent care, a point emphasized by Heng et al. [9]. Our regression analysis revealed that a dentist’s comfort in treating transgender individuals was most strongly predicted by three factors: the inclusivity of their practice environment (β = 0.38), their awareness of gender-affirming clinics (β = 0.29), and their general knowledge of LGBTQIA+ topics (β = 0.22). These findings align with international studies, which show that when providers are trained and supported by inclusive systems, their confidence and competence naturally improve [9,10].

Interestingly, the simple willingness to employ a transgender person did not predict greater clinical comfort. This distinction matters: inclusive hiring is a significant first step, but it does not automatically lead to better patient care. A more holistic approach is needed—one that combines education, awareness, and institutional change. One of the most telling insights from this study is how inclusivity amplifies the effect of knowledge. Our data show that in more inclusive work environments, the benefits of having LGBTQIA+ knowledge are even greater when it comes to hiring and supporting transgender individuals. This reflects a core tenet of the Kartavya Model, proposed by Kumar et al., which advocates for systemic, multi-level reforms encompassing policy, training, and community engagement to create affirming healthcare spaces. This idea also echoes international work by Winter et al., who advocate for comprehensive reforms that integrate cultural competence into every level of the healthcare system [4]. Both frameworks agree: inclusion is not just a checkbox—it is a catalyst for change.

Compared to much of the global literature, this study provides a more optimistic perspective on how Indian dental professionals perceive gender-diverse individuals. Still, there is work to be done. Knowledge gaps, infrastructural shortcomings, and limited clinical experience with transgender patients continue to pose significant barriers. Future efforts must prioritize inclusive curriculum development, structural reforms in practice environments, and sustained community engagement. As highlighted by Pega and Veale, addressing gender identity as a social determinant of health is not just progressive but essential to public health [3].

### 4.1. Positioning Transgender Oral Health Within the Global LGBTQIA+/SGM Equity Paradigm

Although the present study primarily explores transgender inclusivity within oral healthcare, its broader implications extend into the ethical, structural, and public health discourse surrounding equity for LGBTQIA+ and sexual and gender minority (SGM) populations. Across both high- and middle-income contexts, SGM communities continue to face layered forms of exclusion ranging from overt service denial to more insidious institutional invisibility. Practices such as misgendering, heteronormative intake procedures, gender segregated facilities, and inadequate provider competence in affirming communication collectively erode patient trust and discourage engagement with healthcare systems. Even within highly developed health systems, accessibility does not necessarily translate into affirmation. Large-scale studies from Canada and the United States have shown that nearly one in five transgender individuals avoid seeking primary or emergency care due to previous stigma or fear of discrimination [11,12], revealing a paradox in modern healthcare: infrastructure may exist, but empathy often does not.

#### 4.1.1. Global and Comparative Landscape

Cross-national research indicates that structural competence—the capacity of health systems to identify and mitigate social inequities remains underdeveloped in LGBTQIA+ healthcare. Evidence from the United States and Canada, including respondent driven and ethnographic studies, consistently demonstrates that transgender and gender-diverse individuals frequently encounter invalidating documentation, privacy concerns, and practitioners untrained in gender-affirming care [11,12,13]. Even where clinical intent is positive, procedural non-inclusivity such as being misaddressed by pronouns or segregated into binary spaces can generate psychological distress comparable to denial of care.

Within dentistry, these inequities are subtler yet equally significant. U.S. population-based data reveal that sexual and gender minority adults experience approximately 30% higher unmet dental needs than their heterosexual counterparts, alongside poorer self-rated oral health and lower preventive attendance [14]. Minority stress, systemic inflammation, hormonal therapies, and psychosocial adversity converge to increase risks for mucosal lesions, xerostomia, and periodontal disease. Consequently, oral health must be recognized not as peripheral but as an embodied reflection of broader social inequity.

#### 4.1.2. The Indian Experience: Structural, Educational, and Social Determinants

India’s 2011 Census reported roughly 4.9 lakh (0.49 million) transgender individuals, though community based evidence suggests this is a substantial underestimate. Despite progressive policy developments such as the Transgender Persons (Protection of Rights) Act, 2019, structural invisibility persists across healthcare systems. Studies continue to document avoidance of hospitals and clinics among transgender and gender-diverse individuals, driven by fear of ridicule, denial, or breaches of confidentiality [15,16].

In oral health specifically, disparities are further deepened by an absence of empirical data. Few Indian investigations have systematically addressed oral disease profiles, service utilization, or psychosocial determinants among SGM populations. Existing studies often exploratory or attitudinal reveal genuine compassion among dental students and practitioners but limited preparedness to translate empathy into inclusive practice [17]. A landmark global systematic review and meta-analysis found periodontal disease prevalence exceeding 60% among transgender populations, accompanied by elevated caries rates, substance use, and restricted access to care [18], emphasizing the urgent need for gender competent dental frameworks.

#### 4.1.3. Intersectionality and Global Parallels

Oral health occupies a unique intersection of biological, behavioral, and sociocultural determinants, making it a sensitive indicator of structural inequality. In India, the intersecting axes of caste, class, and gender identity compound exclusion a Dalit transgender woman, for example, faces multilayered stigma far exceeding that experienced by her upper-caste or cisgender peers. Comparable dynamics are evident in countries such as Brazil, South Africa, and the Philippines, where socioeconomic vulnerability and institutional discrimination persist despite legislative advances [19]. This global resonance underscores that equitable healthcare cannot be reduced to access alone; it requires recognizing difference as a dimension of dignity rather than deviance.

#### 4.1.4. Toward Affirmative Systems and Curricula

Encouragingly, India’s healthcare education landscape is undergoing reform. The National Medical Commission (2022) incorporated modules on gender diversity and LGBTQIA+ health into the Competency-Based Medical Education framework an important symbolic and pedagogical milestone [20,21]. However, similar curricular integration in dentistry remains limited. Drawing upon models such as Rainbow Health Ontario’s Affirming Care Guidelines [22] and the American Dental Education Association’s Diversity, Equity, and Inclusion (DEI) Checklist [23], dental curricula in India could evolve toward “affirmative competence” where empathy, communication, and inclusive practice are assessed as core clinical skills.

A noteworthy indigenous model is Kartavya—India’s first community-integrated oral-health initiative for transgender persons [24]. Beyond service delivery, Kartavya operationalizes a “care-to-cure” approach by embedding oral healthcare within frameworks of social affirmation, combining preventive care with community engagement and peer-led sensitization of dental professionals.

Within this broader context, the present study’s findings—revealing widespread willingness but inconsistent preparedness among oral healthcare providers—represent a transitional phase rather than a conclusion. They highlight that attitudes, though necessary, must be supported by structured training, inclusive infrastructure, and policy accountability if India’s oral-health system is to truly reflect its constitutional ethos of equality.

#### 4.1.5. Implications for Oral Health Research and the OHP–GSIP © Framework

A metropolitan study from western India provided early empirical insight into dental professionals’ attitudes toward SGM patients [24], reporting generally positive but inconsistent attitudes (mean MCRS = 4.55 ± 1.11). Younger practitioners and those from private institutions exhibited greater empathy compared to their senior or public-sector counterparts. The authors observed that, despite goodwill, the absence of structured LGBTQIA+ training sustains implicit bias and discomfort—mirroring the present study’s finding that acceptance without preparedness remains a principal barrier to inclusive care.

The current study’s results, particularly the strong willingness among oral healthcare professionals to engage with and support transgender individuals, reflect a promising attitudinal base. The OHP–GSIP © instrument can serve as a reliable benchmark for assessing inclusivity, empathy, and preparedness. Expanding its parameters to capture broader SGM experiences—such as perceived stigma, intersectional awareness, and policy responsiveness—could enrich both national and international comparative research.

Future investigations should adopt mixed-method and participatory designs, integrating quantitative indicators with lived narratives. Establishing surveillance systems that collect oral health data disaggregated by gender identity and sexual orientation will be essential to counter invisibility in national databases. Aligning such efforts with India’s National Health Policy (2017) and the UN Sustainable Development Goals (SDGs 3 and 10) would embed oral health firmly within the discourse of social justice and human dignity.

#### 4.1.6. Lacunae in Oral Healthcare and Professional Sensitization

Despite growing visibility of LGBTQIA+ health in medical education, dentistry in India remains relatively unaddressed. While medical curricula increasingly include gender diversity modules, most dental programs continue to operate within binary and heteronormative frameworks, leaving practitioners underprepared to treat SGM patients inclusively. Empirical data on oral-disease burden, behavioral risks, and healthcare utilization among these populations are virtually absent, limited to small-scale attitude surveys. Consequently, oral health remains an overlooked dimension of LGBTQIA+ health inequity, despite its integral link to systemic well-being and psychosocial identity.

Nonetheless, emerging community-led initiatives are bridging this gap. Collaborative workshops and peer-education programs in which transgender advocates co-train dental students have shown notable success in improving empathy, language, and patient interaction. Such experiential models transform awareness into active allyship, challenging stereotypes through direct engagement.

The Kartavya Model [25] exemplifies this approach, integrating preventive care with social participation and peer-led sensitization. By fostering academic community partnerships, Kartavya demonstrates how inclusive oral health delivery can evolve from principle to practice through gender-neutral camps, community-based screening, and referral pathways. Importantly, it redefines members of the transgender community as educators rather than mere beneficiaries, underscoring that empowerment and professional capacity-building can progress simultaneously.

However, such initiatives remain sporadic. Sensitization efforts are often individual-driven rather than institutionalized. Only a few dental schools and clinics have adopted gender-neutral infrastructure or inclusive documentation. Without formal continuing education programs or Dental Council endorsed guidelines, inclusivity remains symbolic rather than systemic. Bridging this divide requires curricular reform, regulatory mandates, and accountability measures that position inclusivity as a measurable competency within professional accreditation frameworks.

#### 4.1.7. Study Limitations and Recommendations

As one of the earliest empirical examinations of gender sensitivity, inclusivity, and preparedness among Indian oral healthcare professionals, this study offers foundational insights but also highlights several methodological considerations. The cross-sectional design provides only a temporal snapshot, without capturing longitudinal changes in attitude or competence. Future cohort-based studies are warranted to evaluate the evolution of inclusivity following educational or policy interventions.

Given its self-reported nature, social desirability bias may have influenced responses, potentially inflating perceived empathy or competence. Subsequent research should integrate objective assessments, such as simulated patient encounters or behavioral evaluations, to reconcile perceived and actual inclusivity.

Although the study achieved broad geographical coverage, its provider-centric focus excluded the lived experiences of transgender and SGM individuals. Incorporating community voices through participatory and qualitative approaches would yield a more balanced understanding of care dynamics. Additionally, including allied dental professionals—such as hygienists, assistants, and administrative staff—would provide a more comprehensive systems-level analysis.

Ultimately, the findings emphasize the need to institutionalize inclusivity through evidence-based educational reforms, standardized training, and measurable competency benchmarks under the Dental Council of India. Linking attitudinal research to policy implementation is essential to transform inclusivity from awareness into sustained professional practice.

#### 4.1.8. Building a Trans-Inclusive Oral Health Ecosystem for India (Table 5)

India is at a pivotal juncture to redefine oral healthcare through a trans-affirmative, evidence driven, and equity-oriented model that integrates research, education, and policy. Incorporating gender identity into national surveys and establishing a Transgender Oral Health Registry could bridge existing data gaps. Employing syndemic and One Health approaches may illuminate the biological and social determinants shaping oral health outcomes.

Peer-led sensitization programs, inclusive curricula, and institutional reforms emphasizing affirming infrastructure and anti-discrimination policies can translate awareness into clinical competence. Furthermore, developing national guidelines, ensuring dedicated funding, and aligning initiatives with the WHO Equity-Based Oral Health Agenda would position India as a global leader in transgender oral health equity where oral healthcare is recognized as both a public health mandate and an act of social justice.

## 5. Conclusions

This study reinforces a key message heard around the world: transgender individuals deserve the same dignity and quality of care as anyone else. Encouragingly, many Indian dental professionals are ready to be part of that change. With proper training, support, and systemic reform, it is possible to create a healthcare system that truly sees and serves everyone—regardless of gender identity. Curriculum modules on gender inclusivity, mandatory sensitivity training, and integration into Dental Council of India accreditation standards should be prioritized.

## Figures and Tables

**Table 1:** Knowledge and Awareness of Gender-Diversity Terms among Dental Professionals (N = 3660).

		% Within Level of Experience			Total (N)	X^2^	*p* Value
UNIT 1: GENDER SENSITIVITY & AWARENESS							
Questions	Options	0–5 years	5–15 years	>15 years			
	Total	100% (n = 1435)	100% (n = 770)	100% (n = 1455)	3660		
1. What is the full form of LGBTQIA?	1. Lesbian, Gay, Bisexual, Transexual, Queer, Intersex, Asexual	67.8% (n = 973)	70.7% (n = 544)	70.2% (n = 1022)	69.4% (n = 2539)	5.932	0.043 *
	2. Lesbian, Gay, Bisexual, Transgender, Queer, Intersex Ally	17.8% (n = 255)	15.8% (n = 122)	15.0% (n = 218)	16.3% (n = 595)		
	3. Both the above	9.8% (n = 141)	9.7% (n = 75)	10.7% (n = 155)	10.1% (n = 371)		
	4. None of the above	4.6% (n = 66)	3.8% (n = 29)	4.1% (n = 60)	4.2% (n = 155)		
2. Does Transgender and Transsexual mean the same?	1. Yes	17.8% (n = 256)	15.8% (n = 122)	15.1% (n = 220)	16.3% (n = 598)	4.083	0.0130 *
	2. No	82.2% (n = 1179)	84.2% (n = 648)	84.9% (n = 1235)	83.7% (n = 3062)		
3. Is Gender and sex the same thing?	1. Yes	15.0% (n = 215)	14.9% (n = 115)	14.6% (n = 213)	14.8% (n = 543)	0.075	0.0491 *
	2. No	85.0% (n = 1220)	85.1% (n = 655)	85.4% (n = 1242)	85.2% (n = 3117)		
4. What do you understand by the term Intersex?	1. When a person’s gender identity or expression do not line up with societal expectation (sex assigned at birth based on genitals)	27.1% (n = 389)	29.4% (n = 226)	27.8% (n = 405)	27.9% (n = 1020)	5.784	0.0448 *
	2. Drag (performance/playing dress-up/cross-dressing)	4.8% (n = 69)	3.5% (n = 27)	4.2% (n = 60)	4.3% (n = 156)		
	3. A general term used for various situations in which a person is born with reproductive or sexual anatomy that does not fit the categories of “female” or “male”.	65.9% (n = 946)	64.0% (n = 493)	65.9% (n = 959)	65.5% (n = 2398)		
	4. Denoting or relating to a person whose sense of personal identity and gender corresponds with their birth sex.	2.2% (n = 31)	3.1% (n = 24)	2.1% (n = 31)	2.3% (n = 86)		
5. What do you understand by the term Cisgender?	1. When a person’s gender identity or expression do not line up with societal expectation (sex assigned at birth based on genitals)	4.9% (n = 71)	4.0% (n = 31)	4.4% (n = 64)	4.5% (n = 166)	5.112	0.0453 *
	2. Drag (performance/playing dress-up/cross-dressing)	8.8% (n = 126)	10.6% (n = 82)	10.9% (n = 159)	10.0% (n = 367)		
	3. A general term used for a variety of situations in which a person is born with reproductive or sexual anatomy that doesn’t fit the categories of “female” or “male”.	8.4% (n = 121)	7.9% (n = 61)	8.3% (n = 121)	8.3% (n = 303)		
	4. Denoting or relating to a person whose sense of personal identity and gender corresponds with their birth sex	77.8% (n = 1117)	77.4% (n = 596)	76.4% (n = 1111)	77.2% (n = 2824)		
6. What do you understand by the term Transgender?	1. When a person’s gender identity or expression does not line up with societal expectations (sex assigned at birth based on genitals)	65.5% (n = 940)	63.5% (n = 489)	67.5% (n = 982)	65.9% (n = 2411)	5.328	0.005 *
	2. Drag (performance/playing dress-up/cross-dressing)	4.0% (n = 58)	4.2% (n = 32)	4.0% (n = 58)	4.0% (n = 148)		
	3. A general term used for a variety of situations in which a person is born with reproductive or sexual anatomy that doesn’t fit the categories of “female” or “male”.	30.4% (n = 436)	32.2% (n = 248)	28.5% (n = 415)	30.0% (n = 1099)		
	4. Denoting or relating to a person whose sense of personal identity and gender corresponds with their birth sex	0.1% (n = 1)	0.1% (n = 1)	0.0% (n = 0)	0.1% (n = 2)		
7. Is gender identity related to gender expression?	1. Yes	39.8% (n = 571)	39.1% (n = 301)	37.1% (n = 540)	38.6% (n = 1412)	2.294	0.0381 *
	2. No	60.2% (n = 864)	60.9% (n = 469)	62.9% (n = 915)	61.4% (n = 2248)		
8. If a person is born as a female but they identify themselves as male, what would be the gender of the person?	1. Male	16.1% (n = 231)	15.5% (n = 119)	17.3% (n = 251)	16.4% (n = 601)		
	2. Female	12.1% (n = 174)	11.6% (n = 89)	12.2% (n = 177)	12.0% (n = 440)		
	3. Gender Fluid	15.9% (n = 228)	18.3% (n = 141)	14.8% (n = 216)	16.0% (n = 585)		
	4. Transgender	55.8% (n = 802)	54.7% (n = 421)	55.7% (n = 811)	55.6% (n = 2034)		
9. When a person wearing a saree walks into your clinic/institute where you work/study, how will you address them?	1. She/Her	27.9% (n = 401)	28.1% (n = 216)	28.0% (n = 408)	28.0% (n = 1025)		
	2. They/Them	4.5% (n = 65)	4.7% (n = 36)	4.3% (n = 62)	4.5% (n = 163)		
	3. Xe/Xem	2.6% (n = 38)	2.2% (n = 17)	2.3% (n = 33)	2.4% (n = 88)		
	4. Ask For Their Preferred Pronouns	64.9% (n = 931)	65.1% (n = 501)	65.4% (n = 952)	65.0% (n = 2384)		
**UNIT 2: SAFE INCLUSIVE SPACES FOR ORAL HEALTHCARE DELIVERY**							
1. In your dental practice or institute in which you work/study, do you find posters, artwork, or information representing the transgender community?	1. Yes	47.2% (n = 678)	43.9% (n = 338)	44.2% (n = 643)	45.3% (n = 1659)	5.430	0.471
	2. No	47.2% (n = 757)	43.9% (n = 432)	44.2% (n = 812)	45.3% (n = 2001)		
2. Do you find any other Information-Education-Communication material on the transgender community in the waiting area of your dental practice or institute you work/study at?	1. Yes	29.2% (n = 419)	29.7% (n = 229)	25.9% (n = 377)	28.0% (n = 1025)	5.329	0.66
	2. No	70.8% (n = 1016)	70.3% (n = 541)	74.1% (n = 1078)	72.0% (n = 2635)		
3. Does your dental practice/institute have a gender-neutral restroom with appropriate symbols/signage?	1. Yes	23.1% (n = 331)	24.0% (n = 185)	23.6% (n = 344)	23.5% (860)	0.285	0.0437 *
	2. No	76.9% (n = 1104)	76.0% (n = 585)	76.4% (n = 1111)	76.5% (n = 2800)		
4. Are genders other than male and female mentioned along with appropriate pronouns for salutations in your dental practice/institute’s intake form (OPD paper) under the gender information section?	1. Yes	17.0% (n = 244)	17.1% (n = 132)	18.1% (n = 263)	17.5% (n = 639)	0.644	0.037 *
	2. No	83.0% (n = 1191)	82.9% (n = 638)	81.9% (n = 1192)	82.5% (n = 3021)		
**UNIT 3: DIVERSITY AND INCLUSIVITY IN DENTAL PRACTICE**							
1. Would you be open to give employment to a transgender person in your dental practice or at the institute you work/study?	1. Yes	89.5% (n = 1284)	90.3% (n = 695)	90.5% (n = 1317)	90.1% (n = 3296)	0.915	0.043 *
	2. No	10.5% (n = 151)	9.7% (n = 75)	9.5% (n = 138)	9.9% (n = 364)		
2. Would you be comfortable working alongside a colleague who identifies themselves as Transgender?	1. Yes	92.0% (n = 1320)	93.5% (n = 720)	92.2% (n = 1341)	92.4% (n = 3381)	1.789	0.0407 *
	2. No	8.0% (n = 115)	6.5% (n = 50)	7.8% (n = 114)	7.6% (n = 279)		
3. Would you be willing to be working with forums working for the development of the transgender community?	1. Yes	94.3% (n = 1353)	93.9% (n = 723)	93.7% (n = 1364)	94.0% (n = 3340)	0.388	0.0482 *
	2. No	5.7% (n = 82)	6.1% (n = 47)	6.3% (n = 91)	6.0% (n = 220)		
4. Would you feel comfortable seeing a person identifying themselves as Transgender in a leadership position at your dental practice/institute you work/study? practice/institute’s intake form (OPD paper) under the gender information section?	1. Yes	95.5% (n = 1370)	96.4% (n = 742)	96.0% (n = 1397)	95.9% (n = 3509)	1.130	0.0456 *
	2. No	4.5% (n = 65)	3.6% (n = 28)	4.0% (n = 58)	4.1% (n = 151)		
**UNIT 4: ATTITUDES OF DENTAL PROFESSIONALS TOWARDS AN INCLUSIVE PRACTICE**							
1. If a person identifying as Transgender seeks treatment at your private practice/institute you work/study at, would you	1. Give them an appointment separately	9.1% (n = 131)	10.0% (n = 77)	9.0% (n = 131)	9.3% (n = 339)	0.645	0.0372 *
	2. Give them an appointment along with other patients	90.9% (n = 1304)	90.0% (n = 693)	91.0% (n = 1324)	90.7% (n = 3321)		
2. If a person identifying as Transgender seeks treatment from your practice/institute you work/study at, would you	1. Do the treatment yourself	94.4% (n = 1354)	94.3% (n = 726)	94.8% (n = 1379)	94.5% (n = 3549)	0.340	0.048 *
	2. Pass it on to your trainees or associate	5.6% (n = 81)	5.7% (n = 44)	5.2% (n = 76)	5.5% (n = 201)		
3. Would you be comfortable treating a transgender person?	1. Yes	89.8% (n = 1288)	87.9% (n = 677)	90.7% (n = 1320)	89.8% (n = 3285)	4.29	0.117
	2. No	10.2% (n = 147)	12.1% (n = 93)	9.3% (n = 135)	10.2% (n = 375)		
4. If a transgender person seeks treatment from your private practice/institute you work/study at, are you likely to give them	1. Discounts if they belong from an economically backward status	67.2% (n = 965)	64.2% (n = 494)	65.6% (n = 955)	66.0% (n = 2414)	2.244	0.0326 *
	2. No discount	32.8%	35.8%	34.4%	34.0%		
5. Befor & after treating a transgender person, would you	1. Use only disposable instruments so that you can throw them away	12.7% (n = 182)	11.7% (n = 90)	11.2% (n = 163)	11.9% (n = 435)	1.548	0.0461 *
	2. Follow appropriate sterilization protocol & reuse non-disposable instruments for other patients	87.3% (n = 1253)	88.3% (n = 680)	88.8% (n = 1292)	88.1% (n = 3225)		
**UNIT 5: ATTITUDES OF DENTAL PROFESSIONALS TOWARDS AN INCLUSIVE PRACTICE**							
1. If a person identifying as Transgender seeks treatment from your private practice/institute you work/study at, you are more likely to check for	1. Common oral manifestations of HIV	10.9% (n = 156)	11.6% (n = 89)	9.9% (n = 144)	10.6% (n = 389)	5.932	0.0431 *
	2. Tobacco history	1.5% (n = 22)	1.4% (n = 11)	2.0% (n = 29)	1.7% (n = 62)		
	3. Hormone replacement therapy	5.8% (n = 83)	7.3% (n = 56)	6.4% (n = 93)	6.3% (n = 232)		
	4. All of the above	81.8% (n = 1174)	79.7% (n = 614)	81.7% (n = 1189)	81.3% (n = 2977)		
2. Are you aware of the nearest testing & treatment facilities for HIV services for referral and linkages?	1. Yes	17.8% (n = 256)	15.8% (n = 122)	15.1% (n = 220)	16.3% (n = 598)	2.711	0.0258 *
	2. No	82.2% (n = 1179)	84.2% (n = 648)	84.9% (n = 1235)	83.7% (n = 3062)		
3. Are you aware of oral manifestations of Hormone Replacement Therapy?	1. Yes	43.8% (n = 628)	45.2% (n = 348)	43.7% (n = 636)	44.0% (n = 1612)	0.525	0.0401 *
	2. No	56.2% (n = 807)	54.8% (n = 422)	56.3% (n = 819)	56.0% (n = 2048)		
4. Do you know about clinics, hospitals and healthcare professionals providing various gender-affirmative healthcare services (in case required by your transgender client)?	1. Yes	27.9% (n = 400)	29.1% (n = 224)	27.6% (n = 401)	28.0% (n = 1025)	0.605	0.0487 *
	2. No	72.1% (n = 1035)	70.9% (n = 546)	72.4% (n = 1054)	72.0% (n = 2635)		

Test applied–Chi-Square (X^2^) test. * indicates statistical significance (*p* < 0.05).

**Table 2:** Descriptive Statistics of Key Variables (N = 3660).

Variable	Mean (SD)	Median (IQR)	Min	Max	Skewness	Kurtosis	Normality (Shapiro–Wilk *p*)
**LGBTQIA Knowledge Score (0–10)**	6.52 (1.78)	7 (5–8)	2	10	−0.46	2.35	<0.001 *
**Comfort in Treating Trans Patients (1–5)**	3.81 (1.09)	4 (3–5)	1	5	−0.52	2.12	<0.001 *
**Awareness of Gender-Affirming Clinics (Yes = 1, No = 0)**	0.55 (0.49)	1 (0–1)	0	1	−0.20	1.04	0.021
**Inclusivity in Practice Setting (1–5)**	3.23 (1.36)	3 (2–4)	1	5	0.14	2.41	0.018
**Willingness to Employ a Trans Person (Yes = 1, No = 0)**	0.62 (0.48)	1 (0–1)	0	1	−0.49	1.13	<0.001 *

* indicates statistical significance (*p* < 0.05).

**Table 3:** Chi-Square Test for Association Between Categorical Variables.

Variables Compared	χ^2^ (df)	*p*-Value	Cramér’s V	Interpretation
**Willingness to Employ a Trans Person vs. Comfort Treating Trans Patients**	0.98 (1)	0.322	0.05	No Significant Association
**Awareness of Clinics vs. Comfort Treating Trans Patients**	25.62 (1)	<0.001 *	0.20	Moderate Association
**Inclusivity in Practice vs. Comfort Treating Trans Patients**	39.24 (1)	<0.001 *	0.27	Strong Association

* indicates statistical significance (*p* < 0.05).

**Table 4:** Multiple Linear Regression Predicting Comfort in Treating Trans Patients. Dependent Variable: Comfort in Treating Trans Patients (1–5 scale).

Predictor Variable	β (Std.)	SE	t-Value	*p*-Value	VIF
**LGBTQIA Knowledge Score**	0.22	0.03	7.11	<0.001 *	1.21
**Inclusivity in Practice Setting**	0.38	0.04	9.32	<0.001 *	1.39
**Awareness of Clinics**	0.29	0.05	6.45	<0.001 *	1.18
**Willingness to Employ a Trans Person**	0.07	0.06	1.45	0.147	1.05

* indicates statistical significance (*p* < 0.05).

**Table 5:** Building a Trans-Inclusive Oral Health Research and Policy Ecosystem for India.

Strategic Domain	Key Focus Areas	Proposed Actions/Research Directions	Expected Outcomes
A. Research and Epidemiological Frameworks	Population-Level Surveillance	Integrate gender identity and sexual orientation variables within the National Oral Health Programme and NFHS surveys.	Enables disaggregated disease surveillance, fair allocation of resources, and responsive policy making.
	Longitudinal Impact Research	Conduct cohort-based and bio-behavioral investigations on the long-term oral and craniofacial effects of hormone therapy, surgeries, and psychosocial distress.	Determines cause effect pathways between gender-affirming care, psychological stress, and oral-systemic health.
	Mixed-Methods and Geo-Syndemic Approaches	Utilize syndemic and spatial epidemiology to analyze oral–mental health–substance use intersections and regional exclusion gradients.	Offers comprehensive understanding of layered determinants and health disparities
	Biopsychosocial Integration in Research	Link laboratory, behavioral, and clinical sciences to evaluate inflammatory biomarkers and oral–systemic pathways within One Health paradigms.	Enhances evidence for biological and psychosocial relationship and situates dentistry in precision public health.
	Indian Transgender Oral Health Database	Structure a national anonymized registry with collaboration from community led organizations (e.g., Kartavya Disha Global Foundation).	Build an ethical and continuous data platform supporting evidence driven public health policies.
B. Translational and Implementation Research	Intervention Trials	Assess the effect of peer-led sensitization programs, digital education modules, and inclusive clinical redesigns through pre–post and cluster RCT frameworks.	Evaluates practical success of inclusivity strategies on patient satisfaction, service quality, and health outcomes.
C. Educational Transformation	Curricular Embedding	Make LGBTQIA+/SGM sensitization modules mandatory at UG/PG levels as a part of DCI’s competency standards.	Establishes inclusivity as an assessed professional standard.
	Faculty Capacity Building	Establish Train-the-Trainer centres for inclusive pedagogy, mentorship, and gender-sensitive communication skills.	Creates sustainable educator networks and strengthens cultural understanding.
	Simulation and Immersive Learning.	Introduce standardized transgender patient scenarios and community immersion postings.	Converts empathy into experiential competence and measurable behavioral transformation.
D. Clinical and Institutional Transformation	Inclusive Infrastructure	Provides incentives to dental facilities and institutions for adopting gender-neutral restrooms, inclusive documentation, signage, and diverse visual representation.	Enhances patient comfort, promotes trust, and ensures readiness of clinics for diverse clients.
	Safe Spaces & Grievance Protocols	Incorporate anti-discrimination, confidentiality, and dignity clauses within institutional QA systems (NABH/NABL).	Strengthens accountability while embedding respect within institutional culture.
	Community Partnerships	Establish collaborations with transgender welfare boards, CBOs, and NGOs for outreach and oral-health screening.	Deepens community trust, promotes co-learning, and supports grassroots empowerment.
E. Policy and Systems Transformation	National Policy Standards	Frame National Guidelines for Gender-Inclusive Oral Healthcare under MoHFW.	Ensures uniform care delivery and ethical behaviour across clinical and academic institutions.
	Grant Prioritization	Encourage ICMR, DBT, DST to support research projects on SGM oral health, stigma, and policy inclusion.	Expands the research base and promotes innovation through translational funding.
	International Collaborations	Coordinate with Indian initiatives with WHO’s Equity and Rights-Based Oral Health Agenda 2030 and global SGM health frameworks.	Facilitates reciprocal learning and global benchmarking in inclusive oral healthcare.

## Data Availability

Data may be made available from the corresponding authors upon reasonable request and with prior approval from the Institutional Ethics Committee.

## References

[1] (2011). Census of India.

[2] Government of India (2019). The Transgender Persons (Protection of Rights) Act.

[3] Pega F., Veale J.F. (2015). The case for the World Health Organization’s Commission on Social Determinants of Health to address gender identity. Am. J. Public Health.

[4] Winter S., Settle E., Wylie K., Reisner S., Cabral M., Knudson G., Baral S. (2016). Synergies in health and human rights: A call to action to improve transgender health. Lancet.

[5] Reisner S.L., White J.M., Bradford J.B., Mimiaga M.J. (2016). Transgender health disparities: Comparing full cohort and nested matched-pair study designs in a community health center. LGBT Health.

[6] James S.E., Herman J.L., Rankin S., Keisling M., Mottet L.A., Anafi M. (2016). The Report of the 2015 U.S. Transgender Survey.

[7] Armuand G.M., Dhejne C., Olofsson J.I., Stefenson M. (2017). Transgender people’s experiences of fertility preservation: A qualitative study. Hum. Reprod..

[8] Obedin-Maliver J., Goldsmith E.S., Stewart L., White W., Tran E., Brenman S., Wells M., Fetterman D.M., Garcia G., Lunn M.R. (2011). Lesbian, gay, bisexual, and transgender–related content in undergraduate medical education. JAMA..

[9] Heng J.Y., Hackett J., Minchin K. (2018). Dental students’ and dental professionals’ attitudes towards LGBT patients: A scoping review. J. Dent. Educ..

[10] Safer J.D., Tangpricha V. (2019). Care of transgender persons. N. Engl. J. Med..

[11] Bauer G.R., Scheim A.I., Deutsch M.B., Massarella C. (2015). Reported emergency department avoidance, use, and experiences of transgender persons in Ontario, Canada: Results from a respondent-driven sampling survey. Ann. Emerg. Med..

[12] Sanchez N.F., Sanchez J.P., Danoff A. (2009). Health care utilization, barriers to care, and hormone usage among male-to-female transgender persons in New York City. Am. J. Public Health.

[13] Nemoto T., Bödeker B., Iwamoto M. (2011). Social support, exposure to violence, and transphobia among transgender women in the San Francisco Bay Area. Am. J. Public Health.

[14] Schwartz S.B., Sanders A.E., Lee J.Y., Divaris K. (2019). Sexual-orientation–related oral-health disparities in the United States. J. Public Health Dent..

[15] Chakrapani V., Newman P.A., Shunmugam M., Logie C.H., Samuel M. (2017). Syndemic and intersectional stigma among HIV-positive men who have sex with men and transgender women in India. Soc. Sci. Med..

[16] Scheim A.I., Coleman T., Lachowsky N., Bauer G.R. (2021). Health care access among transgender and nonbinary people in Canada, 2019: A cross-sectional survey. CMAJ Open.

[17] Sharma S., Shukla S., Kamate S.K., Walia S., Kumari S., Jain M., Kalsi R. (2023). An exploratory research comparing oral health, pattern of substance abuse and nicotine dependence among LGBT, female sex workers and heterogenders. J. Contemp. Dent. Pr..

[18] Kumar V., Thakker J., Royal A., Bhanushali N., Baghdadi Z.D. (2023). Oral health and hygiene status of global transgender population: A living systematic review and meta-analysis. Int. J. Environ. Res. Public Health.

[19] Puhl R.M., Brownell K.D. (2006). Confronting and coping with weight stigma: An investigation of overweight and obese adults. Obesity.

[20] National Medical Commission (NMC) (2022). Competency-Based Curriculum for the Indian Medical Graduate.

[21] Dandekar S.P., Mahdi F., Chacko T.V. (2023). A Critical Appraisal of the New Competency-Based Medical Undergraduate Curriculum in Biochemistry. Ind. J. Clin. Biochem..

[22] Bourns A. (2021). Guidelines for Gender-Affirming Primary Care with Trans and Non-Binary Patients.

[23] American Dental Education Association (ADEA) (2020). Checklist to Promote Diversity, Equity and Inclusion.

[24] Kumar V., Atre S., Jain R., Bhanushali N., Singh S., Chaudhari S. (2021). Include-integrate-involve: Deciphering oral healthcare providers’ professional demeanor towards sexual and gender minority cohorts in a metropolitan city of western India. J. Oral. Biol. Craniofac Res..

[25] Kumar V., Jain R., Singh S. (2021). Kartavya: An innovative model to deliver oral health services to transgender community in India. Spec. Care Dent..

